# Lessons we learn from review of urological procedures performed during three decades in a spinal cord injury patient: a case report

**DOI:** 10.1186/1757-1626-2-9334

**Published:** 2009-12-16

**Authors:** Subramanian Vaidyanathan, Bakul M Soni, Peter L Hughes, Gurpreet Singh, Paul Mansour, Tun Oo

**Affiliations:** 1 Spinal Injuries Unit, District General Hospital, Town Lane, Southport PR8 6PN, UK; 2 Department of Radiology, District General Hospital, Southport PR8 6PN, UK; 3 Department of Urology, District General Hospital, Southport PR8 6PN, UK; 4 Department of Cellular Pathology, District General Hospital, Southport PR8 6PN, UK

## Abstract

**Background:**

We review urological procedures performed on a spinal cord injury patient during three decades.

**Case presentation:**

A 23-year-old male patient sustained T-12 paraplegia in 1971. In 1972, intravenous urography showed both kidneys functioning well; division of external urethral sphincter was performed. In 1976, reimplantation of left ureter (Lich-Gregoir) was carried out for vesicoureteric reflux. As reflux persisted, left ureter was reimplanted by psoas hitch-Boari flap technique in 1978.

This patient suffered from severe pain in legs; intrathecal injection of phenol was performed twice in 1979. The segment bearing the scarred spinal cord was removed in September 1982.

This patient required continuous catheter drainage. Deep median sphincterotomy was performed in 1984. As the left kidney showed little function, left nephroureterectomy was performed in 1986. In an attempt to obviate the need for an indwelling catheter, bladder neck resection and tri-radiate sphincterotomy were carried out in 1989; but these procedures proved futile. UroLume prosthesis was inserted and splinted the urethra from prostatic apex to bulb in October 1990. As mucosa was apposing distal to stent, in November 1990, second UroLume stent was hitched inside distal end of first. In March 1991, urethroscopy showed the distal end of the distal stent had fragmented; loose wires were removed. In April 1991, this patient developed sweating, shivering and haematuria. Urine showed Pseudomonas. Suprapubic cystostomy was performed. Suprapubic cystostomy was done again the next day, as the catheter was pulled out accidentally during night. Subsequently, a 16 Fr Silastic catheter was passed per urethra and suprapubic catheter was removed. In July 1993, Urocoil stent was put inside UroLume stent with distal end of Urocoil stent lying free in urethra. In September 1993, this patient was struggling to pass urine. Urocoil stent had migrated to bladder; therefore, Urocoil stent was removed and a Memotherm stent was deployed. This patient continued to experience trouble with micturition; therefore, Memotherm stent was removed. Currently, wires of UroLume stent protrude in to urethra, which tend to puncture the balloon of urethral Foley catheter, especially when the patient performs manual evacuation of bowels.

**Conclusion:**

We failed to implement intermittent catheterisation along with anti-cholinergic therapy. Instead, we performed several urological procedures with unsatisfactory outcome; the patient lost his left kidney. We believe that honest review of clinical practice will help towards learning from past mistakes.

## Background

Discussions about medical errors facilitate professional learning for physicians [1]. We reviewed urological procedures, which were carried out on a spinal cord injury patient during three decades, in order to identify our mistakes and learn from past experience.

## Case presentation

A British, Caucasian male patient, born in 1948, was working as a bricklayer. He was painting on 10 December 1971; the wind was blowing strongly, he lost his balance and fell from the ladder about 22 feet. He was unable to feel or move his legs. X-ray of lumbar spine showed wedge compression of T-12. Clinical examination revealed complete loss of power in all groups of muscles and sensation to pinprick and light touch at L-2 and below. Bed rest was the mainstay of treatment for fracture of thoracic spine. He was advised intermittent catheterisation. Intravenous urography, performed on 13 March 1972, showed no radio opaque calculi. Both kidneys were functioning; left renal tract was normal. Right kidney was partially unascended, rotated, and slightly hydronephrotic.

**Figure 1:**
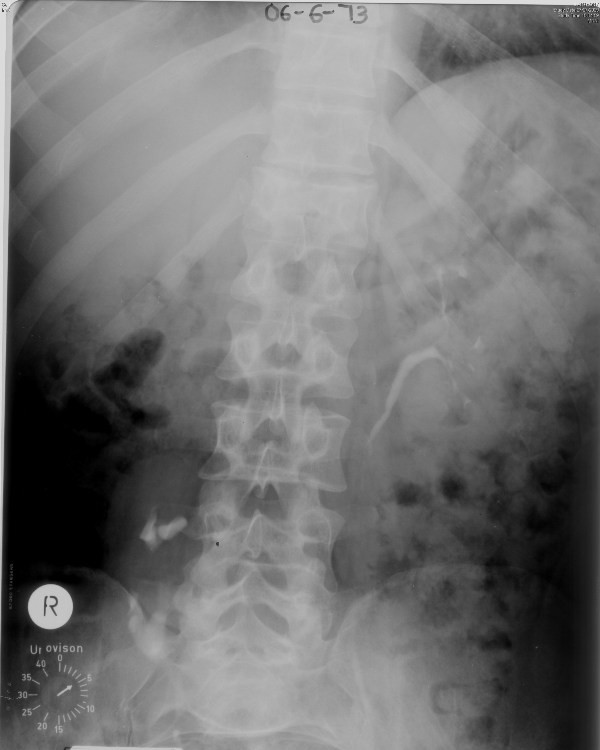
**Intravenous urography (06 June 1973): Five minutes film shows normal left kidney with undilated pelvicalyceal system and ureter**. Right kidney is located at L4-5 level and malrotated.

Postero-bilateral division of external urethral sphincter was performed on 11 April 1972. Following this procedure, he had to strain or lift himself to pass urine. Intravenous urography, performed on 06 June 1973, showed no dilatation of upper urinary tracts (Figure 1). Intravenous urography of 14 July 1975 showed function in both kidneys. But the bladder showed *increased trabeculation and diverticular formation*.

Cystogram of 08 October 1975 revealed reflux into left ureter. He was getting repeated urine infections. He was prescribed antibacterials, alternating fortnightly courses of nalidixic acid and ampicillin. Reimplantation of left ureter was carried out on 17 February 1976. Lich-Gregoir extravesical ureteric tunnelling was done. Follow-up cystogram performed on 07 December 1977, showed reflux up the whole of left ureter. Urine microbiology showed Pseudomonas species. On 03 February 1978, left ureter was reimplanted by psoas hitch-Boari flap technique. Cystogram on 28 June 1978 showed reflux in to lower ureter at the end of micturition.

This patient was suffering from severe pain in the legs. Intrathecal injection of phenol in glycerine was performed on 09 and 27 February 1979.

Intravenous urography, performed on 06 November 1979, showed bilateral hydronephrosis. There was very marked deformity of urinary bladder with diverticulum formation. In January 1981, intravenous urography showed *hydronephrosis of left kidney with rather poor function*; right kidney was a little hydronephrotic.

He was suffering from pain in left adductor region and right buttock. Lumbar sympathetic block was carried out on 05 August 1982 with 15 ml of aqueous phenol. There was exacerbation of pain following sympathetic block, spreading to right side around T-11/12. In September 1982, the segment bearing the scarred spinal cord was removed with laser. Unfortunately, pain was quite severely exacerbated for ten days. The neurosurgeon waited for a few weeks to see if there would be a late response to cord section but, unfortunately, there was not. Therefore, on 18 September 1982, using a segment of much more normal cord above the level of section, eight paired dorsal root entry zone lesions were performed using thermocoagulator and a cordotomy electrode. This of course was done under direct vision by open operation. Following this, the residual pain was very acceptable and tolerable compared with what he had experienced originally.

In August 1983, this patient developed severe urine infection while on holiday. Intravenous urography, performed in a local hospital, showed bilateral hydronephrosis. He required continuous catheter drainage. In January 1984, deep median sphincterotomy was performed. The next day after this procedure, patient developed high fever. Urine showed *Pseudomonas aeruginosa *and he was prescribed netilmycin 125 mg twice a day intravenously. Although he was quite well for a week after discharge, he was in trouble again; he developed sweating, rigors and passed bloodstained urine. He was prescribed long-term nitrofurantoin.

**Figure 2:**
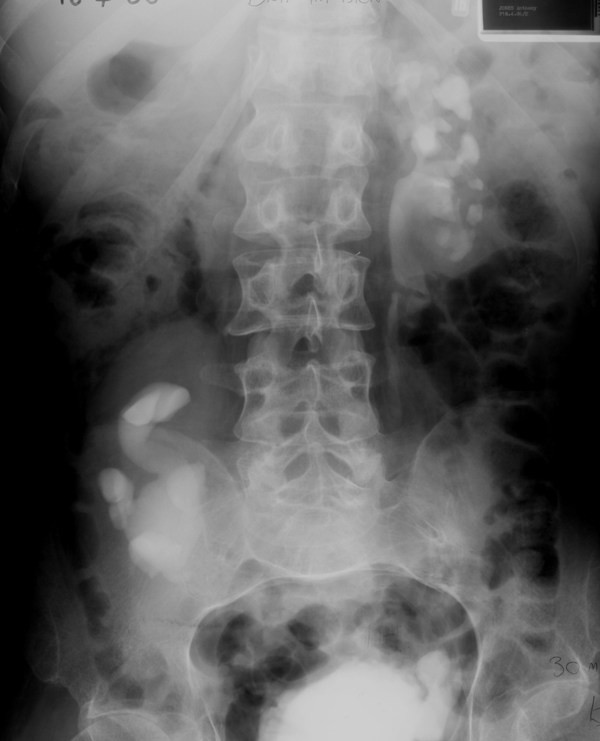
**Drip infusion pyelography (18 April 1986) shows clubbing of calyces and thinning of left renal cortex**. There is dilatation of the pelvicalyceal system in the malrotated right kidney.

In April 1986, intravenous urography right kidney to be functioning well; left kidney showed clubbing of calyces and thinning of renal cortex. (Figure 2) Left nephroureterectomy was performed on 28 November 1986. Urethral catheter drainage was being continued. Bladder neck resection and triradiate sphincterotomy was carried out on 24 November 1989. In March 1990, this patient continued to have indwelling urethral catheter and was experiencing recurrent urine infections.

On 26 October 1990, 3 cm UroLume spiral prosthesis was inserted and splinted the area from prostatic apex to bulb. Urethroscopy, performed on 23 November 1990, showed stent in position, but mucosa was opposing distal to stent. Therefore, second UroLume stent was hitched inside distal end of first and deployed. Urethroscopy, performed on 22 March 1991, showed the distal end of the distal stent had fragmented with a few loose ends spiking the urethra; the rest had epithelialised. All loose ends were removed, thus providing free access to bladder, which was clear.

**Figure 3:**
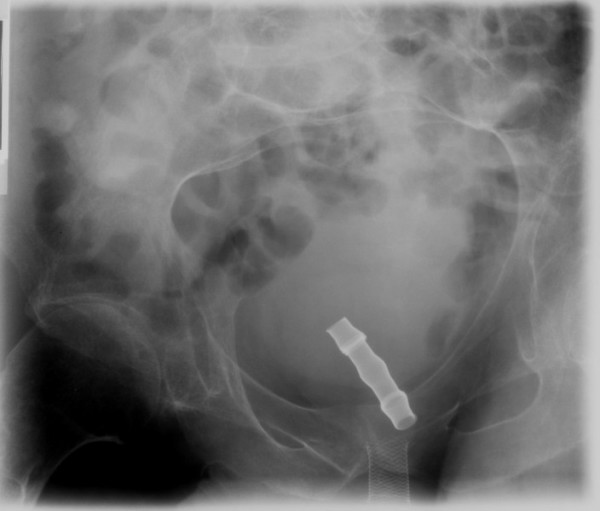
**Cystogram (10 September 1993) shows Urocoil stent has migrated proximally into urinary bladder**.

This patient was admitted to spinal unit on 18 April 1991 with sweating, shivering and mild haematuria. Urine showed Pseudomonas. Suprapubic cystostomy was performed. On 19 April 1991, suprapubic cystostomy was performed again as the catheter got pulled out accidentally in the night. Urethroscopy, performed on 11 May 1991, showed distal few strands of the Wall stent were still protruding and were removed. The proximal stent had epithelialised nicely. A 16 Fr Silastic catheter was passed with ease; suprapubic catheter was removed. Cystoscopy, performed on 30 August 1992, showed the stents were covered generally except for a few strands anteriorly. Granulations were seen in one or two places but otherwise, the urethra was open. But this patient continued to suffer from recurrent urine infections with Pseudomonas. On 09 July 1993, urethroscopy showed few terminal wires from distal stent spiking; the proximal stent was nicely in position with epithelialised urethra and no overgrowth. Urocoil stent was put within UroLume stent with distal end of Urocoil stent lying free in capacious urethra. In September 1993, this patient was feeling no better; he was struggling to pass urine. X-ray of pelvis and cystogram revealed that Urocoil stent had migrated to bladder (Figure 3). It was decided to remove Urocoil stent and insert Memotherm stent. On 12 November 1993, 80 cm, 42 Ch Memotherm stent was deployed within distal end of UroLume and settled nicely into the urethra across the narrow segment. This patient started experiencing difficulties in passing urine. On 04 February 1994, urethroscopy revealed that an angle had formed at the distal end of the Memotherm with granulations. The problem was that an angle would always tend to form if the distal stent were to lie in the bulbous urethra. The further option, to avoid a catheter, would be to put a stent extending distally. On 24 March 1994, urethroscopy was performed because of trouble with micturition and no catheter could be passed. Urethroscopy revealed encrustations and generous mucosal overgrowth. Therefore, the Memotherm stent was removed.

**Figure 4:**
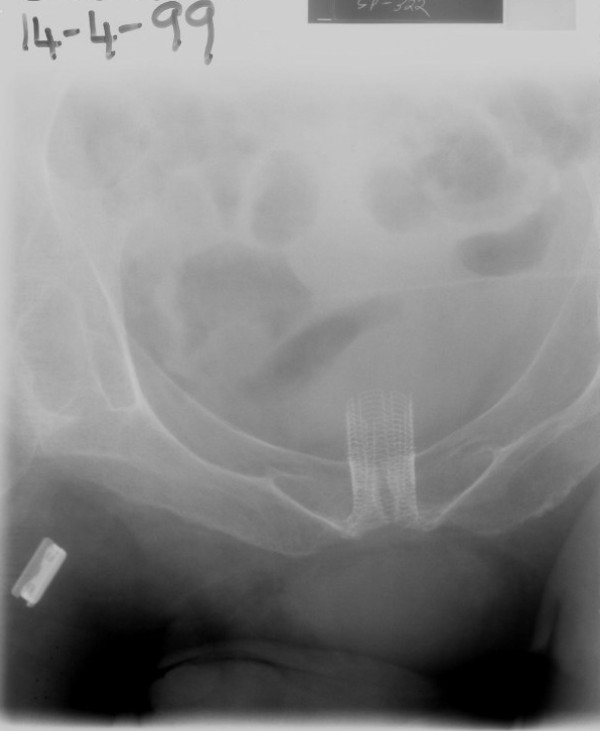
**X-ray of urinary bladder (14 April 1999) shows UroLume stent**. Urocoil stent had been removed.

In May 1996, this patient was admitted with chills and kidney pain. He was given gentamicin and his condition improved. On 03 June 1996, urine showed Pseudomonas resistant to gentamicin, piperacillin and ciprofloxacin. He was advised intermittent catheterisation. He was doing three catheters a day but it became increasingly difficult to do a catheter through UroLume stent. (Figure 4) Cystoscopy, performed on 23 December 2002, showed metallic wires protruding in to the urethra. There was angulation of stent with a cul de sac. A 0.032" guide wire was passed in to urinary bladder; a 14 Fr. Foley catheter was inserted over the guide wire.

**Figure 5:**
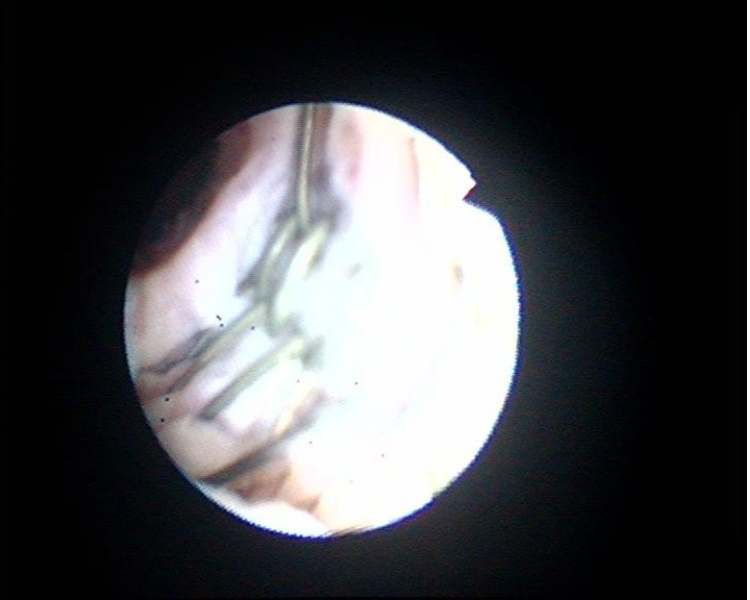
**Flexible cystoscopy shows naked wires of UroLume stent**. These wires are not covered by urethral mucosa.

**Figure 6:**
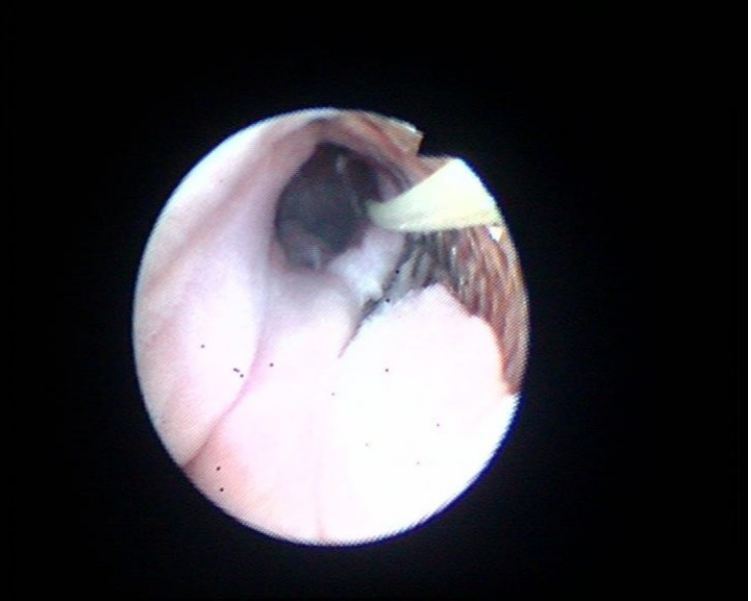
**Flexible cystoscopy shows a guide wire, which has been introduced through the stent**. Exposed wires of UroLume stent are clearly visible by the side of guide wire.

**Figure 7:**
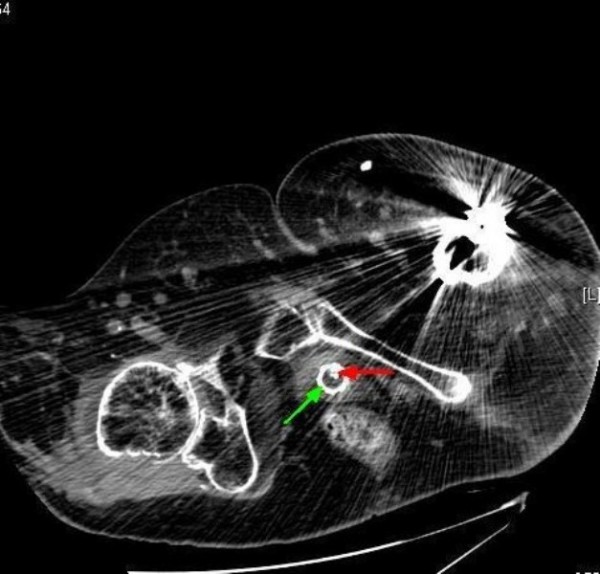
**Computed tomography of pelvis (24 February 2009): axial section shows circular ring of stent located just behind symphysis pubis (green arrow); a wire is protruding into the lumen (red arrow)**.

**Figure 8:**
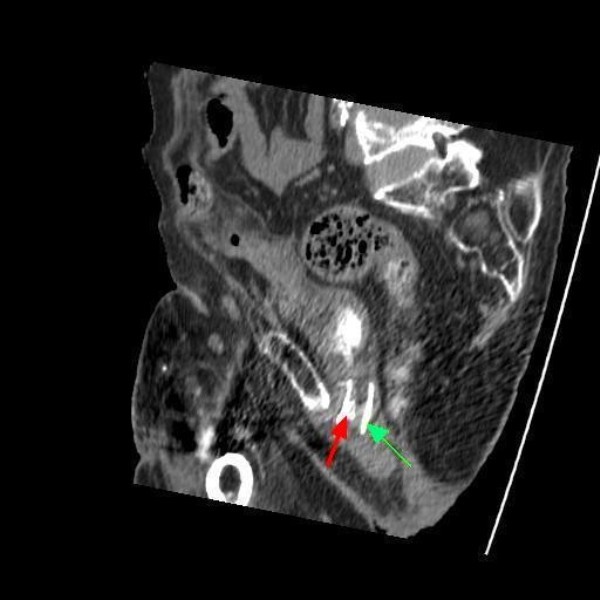
**Computed tomography of pelvis (24 February 2009): sagittal section shows the stent in its long axis (green arrow) along with a wire protruding into the lumen of stent (red arrow)**.

At present, flexible cystoscopy shows wires of stent protruding in to urethra. (Figure 5) This patient continues to have 16 Fr Foley catheter inserted over a guide wire. (Figure 6) Occasionally, the protruding wires of UroLume stent puncture the balloon of Foley catheter, especially when the patient performs manual evacuation of bowels. This leads to extrusion of Foley catheter from urinary bladder and the patient has to rush to the hospital for catheterisation with flexible cystoscopy. Wires protruding in to lumen of urethra are seen clearly in computed tomography of pelvis, which was performed on 24 February 2009. (Figures 7 and 8)

**Figure 9:**
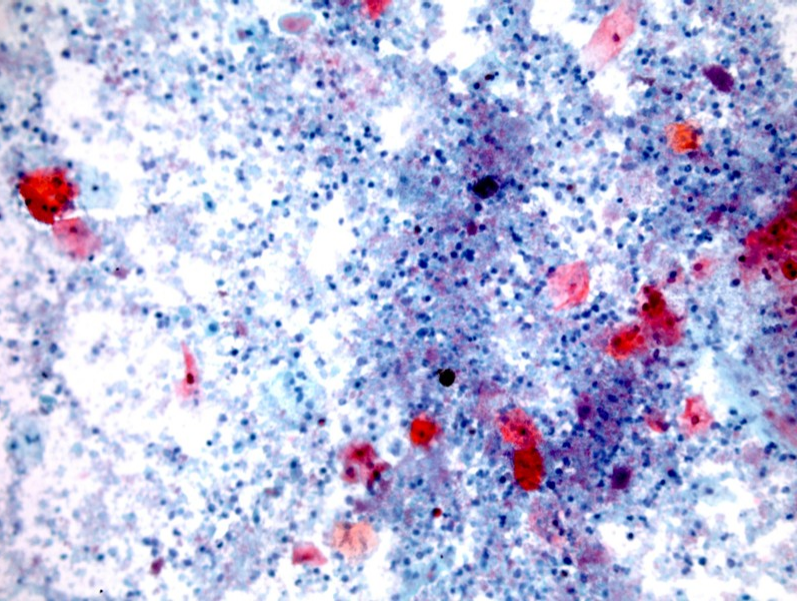
**Urine cytology specimen (Papanicolau stain) shows scattered, benign, squamous epithelial cells with bright red/orange cytoplasm suggesting keratinising squamous metaplasia, against an inflammatory background of polymorphs and debris**.

**Figure 10:**
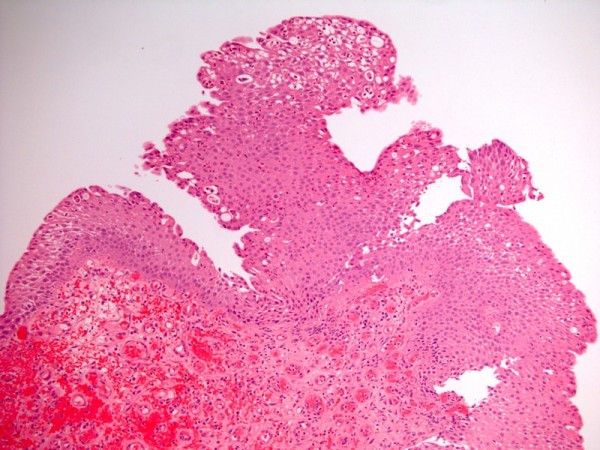
**Bladder biopsy specimen (H&E stain) showing intensely congested mucosa (below) bearing a polypoid projection of inflamed, benign urothelium (top), warranting a diagnosis of polypoid cystitis**.

Cytology of urine showed scattered, benign, squamous epithelial cells with bright red/orange cytoplasm suggesting keratinising squamous metaplasia. (Figure 9) Biopsy of bladder mucosa revealed intensely congested mucosa bearing a polypoid projection of inflamed, benign urothelium, warranting a diagnosis of polypoid cystitis. (Figure 10)

## Discussion

Spinal cord injury affects renal function. A decrease in glomerular filtration rate is seen in patients with cervical and thoracic levels of injury and complete injuries. Improvement in renal function occurs most clearly in patients who use clean intermittent catheterisation as a bladder emptying method [2]. In this patient, we failed to implement intermittent catheterisation along with anticholinergic therapy. Instead, this patient underwent sphincterotomy three times; UroLume, Urocoil and Memotherm stents were inserted. Ultimately, this patient underwent left nephrectomy and required long-term indwelling catheter drainage.

We learn from this case that health professionals should make serious efforts to institute intermittent catheterisation along with anticholinergic therapy in spinal cord injury patients. Had we implemented intermittent catheterisation regime, all the urological procedures could have been avoided and the patient would not have lost his left kidney.

In order to prevent mistakes in diagnosis and to detect medical errors without delay, it has been recommended that physicians set aside time *to reflect upon their clinical practice*, regularly participate in honest and informal case discussions, and seek a second opinion when in doubt [3].

## Conclusion

Doctors should make every possible effort to institute intermittent catheterisation along with anticholinergic therapy in spinal cord injury patients with neuropathic bladder. Sphincterotomy is unlikely to be effective in patients, who had undergone intrathecal injection of phenol or cordectomy. We believe that honest review of clinical practice will help doctors to learn from past mistakes.

## Patient's perspective

I am a married male, who has been a Paraplegic for 38 years. Renal problems have always been the foremost. I had a nephrectomy on my left side. I had high residuals in my bladder in late 1980's and it was decided that I required a stent. The three types of stent did not work. Over several years, the endourethral wall stent had deteriorated, more so during last four years. It is possible for me to see, during cystoscopy, wires of the stent sticking out into the bladder neck. Wires can burst the balloon of Foley catheter. There is no set day, night, or time for this occurring, which results in me having to go and have a new catheter fitted at the spinal unit (without whose help none of this would be possible). I question if many patients are stoic enough to live with this.

I take Ciprofloxacin 500 mg twice a day when the catheter is changed to combat infections. But sometimes, I require antibiotics to be given intravenously; then I am admitted to spinal unit. Would I choose to have a stent today? I would so definitely "NO".

## Consent

Written informed consent was obtained from the patient for publication of this case report and accompanying images. A copy of the written consent is available for review by the Editor-in-Chief of this journal.

## Competing interests

The authors declare that they have no competing interests.

## Authors' contributions

SV performed flexible cystoscopy, developed the concept and wrote draft; BMS was the Consultant in charge of this patient; PH reviewed medical images; GS performed bladder biopsy; PM examined bladder biopsy and urine cytology; TO provided clinical care. All authors read and approved the final manuscript.

## References

[B1] Kaldjian LC Forman-Hoffman VL Jones EW Wu BJ Levi BH Rosenthal GE Do faculty and resident physicians discuss their medical errors? J Med Ethics 2008 34 10 717 722 10.1136/jme.2007.023713 18827101

[B2] Pettersson-Hammerstad K Jonsson O Svennung IB Karlsson AK Impaired renal function in newly spinal cord injured patients improves in the chronic state effect of clean intermittent catheterization? J Urol 2008 180 1 187 191 10.1016/j.juro.2008.03.051 18499190

[B3] Vaidyanathan S Soni BM Hughes PL Mansour P Singh G Pitfalls in radiologic and histopathologic diagnosis of urologic disease report of 4 cases Adv Ther 2006 23 6 1030 1039 10.1007/BF02850223 17276970

